# *Salmonella* Bredeney Infections Linked to a Brand of Peanut Butter — United States, 2012

**Published:** 2013-02-15

**Authors:** J. Kathryn MacDonald, Ernest Julian, Alvina Chu, Jamie Dion, William Boden, Stelios Viazis, Jennifer Beal, William Lanier, Thai-An Nguyen, Laura Burnworth, Ian Williams, Laura Gieraltowski, W. Thane Hancock

**Affiliations:** Washington State Dept of Health; Rhode Island Dept of Health; Maryland Dept of Health and Mental Hygiene; Denver District Office; Coordinated Outbreak Response and Evaluation Network, Food and Drug Administration; Div of Foodborne, Waterborne, and Environmental Diseases, National Center for Emerging and Zoonotic Infectious Diseases; EIS Officer, CDC

In 2012, CDC collaborated with state health and agricultural agencies and the Food and Drug Administration (FDA) to investigate an outbreak of *Salmonella* Bredeney infections associated with exposure to peanut products manufactured by Sunland, Inc. of Portales, New Mexico (1).

On August 23, 2012, CDC PulseNet, a molecular subtyping network for foodborne disease surveillance, reported a cluster of 14 *Salmonella* Bredeney infections with an indistinguishable pulsed-field gel electrophoresis (PFGE) pattern. This PFGE pattern is rare, with five to eight reports uploaded to PulseNet annually (1). *Salmonella* Bredeney is a rare serotype, with only one documented outbreak in the United States before 2012.

During June 11–November 8, 2012, a total of 41 cases of *Salmonella* Bredeney infections were identified in 20 states. The median age of patients was 6 years (range: <1–79 years); 63% of patients were aged <10 years, and 60% were male. Among 36 patients for whom information was available, 10 (28%) were reported to have been hospitalized. No deaths have been reported.

Of the 32 patients for whom information was available, 25 (78%) had eaten a Trader Joe’s brand Valencia peanut butter product manufactured by Sunland, Inc. Testing conducted by the New Jersey Department of Health, Virginia Division of Consolidated Laboratory Services, and Washington State Department of Agriculture laboratories isolated the outbreak strain of *Salmonella* Bredeney from three opened jars of Trader Joe’s Creamy Salted Valencia Peanut Butter collected from three different patients’ homes.

During September 17–October 16, 2012, FDA conducted an inspection of Sunland, Inc. manufacturing facilities (2). Environmental samples and samples from unopened peanut butter jars collected by FDA from the nut butter production facility yielded the outbreak strain of *Salmonella* Bredeney.

On September 24, 2012, Sunland, Inc. announced a voluntary recall of almond butter and peanut butter products manufactured in the Sunland, Inc. nut butter production facility during May 1–September 24, 2012. On October 4, 2012, Sunland, Inc. expanded its recall to include all products made in its nut butter production facility during March 1, 2010–September 24, 2012. On October 12, 2012, Sunland, Inc. extended the voluntary recall to include raw and roasted shelled and in-shell peanuts processed in its peanut processing plant (2). Approximately 300 products have been recalled (3).

CDC recommends that consumers not eat recalled Sunland, Inc. products or foods containing recalled products and discard or return any remaining recalled products.
